# *Vitis vinifera* Production in Michigan: Factors and Trends Driving Cultivation Patterns

**DOI:** 10.3389/fpls.2021.704690

**Published:** 2021-07-06

**Authors:** Erin L. Bunting, Dan Wanyama, Robert Goodwin, Nicholas Weil, Paolo Sabbatini, Jeff Andresen

**Affiliations:** ^1^Department of Geography, Environment, and Spatial Sciences, Michigan State University, East Lansing, MI, United States; ^2^Remote Sensing and GIS Research and Outreach Services, Michigan State University, East Lansing, MI, United States; ^3^Department of Horticulture, Michigan State University, East Lansing, MI, United States

**Keywords:** *Vitis vinifera*, Michigan, climate change, temperature, precipitation, viticulture, wine grapes, trend analysis

## Abstract

Vinifera cultivation is a thriving and growing industry across the state of Michigan (MI), United States. Extensive time, funds, and effort have been applied by the industry to promote growth and the onset of new producers. Specifically, *Vitis vinifera* wine grapes, which have been cultivated in MI since the 1970s, have seen a rapid expansion and investment from both first-time and legacy growers. However, historically, the climate of MI presented a challenge for cultivation because of low growing season temperatures (GSTs), short growing seasons, and excessive precipitation at the time of harvest. Over time, two key factors have led the MI wine industry to overcome the challenging climate. First, as seen in the literature, there are noted changes in climate, especially since the late 1980s, leading to more favorable conditions for cultivation. Second, MI growers traditionally focused on *V. vinifera* cultivation, which is susceptible to low winter temperatures, selected less vulnerable regions within the state while also focusing on vine protection techniques. Given the rapid growth of the wine industry across MI, there is a need to understand suitability and its drivers to help all growers make economically impactful decisions on production and expansion of wine grapes. This article looked to study the suitability of MI *vinifera* across the state in two ways. Initially, through an extensive literature review, the key drivers and commonly noted trends guiding vinifera production were chronicled. Second, through a trend analysis of the key drivers of suitability, the study investigated how such variables are changing significantly over space and time. The results of this study expand the knowledge of cool climate agriculture production and suitability for cultivation and highlight the complexity of relating suitability drivers for non-cool climate to cool climate vinifera cultivation.

## Introduction

Climate change is a major environmental concern today, because it has introduced critical changes to ecosystems of the Earth (Arora, 2019). While climate change is not a new phenomenon, the climate system is changing more rapidly than in the past, primarily because of increased greenhouse gas emissions into the atmosphere (Duchene and Schneider, 2005; IPCC, 2007; Aydinalp and Cresser, 2008; Nelson et al., 2009; Arora, 2019). As a result, temperatures have increased significantly (Duchene and Schneider, 2005; IPCC, 2007; Arora, 2019), precipitation patterns have been altered, extreme natural events (such as droughts, floods, and heat waves) have intensified (IPCC, 2007; Arora, 2019), seasons have shifted (Nelson et al., 2009), and degraded land has significantly expanded over time (Arora, 2019). These effects have manifested in many important socio-economic sectors like public health, water, and agriculture. Agriculture depends heavily on weather and climate conditions to support crop growth and development. For production to be successful, favorable climate conditions should persist at all stages of the life cycle of crops. This is attributed to the fact that many crops are affected by both mean climate conditions and the incidence of extreme weather events at key stages of development (Moriondo and Bindi, 2006; Beacham et al., 2018), making agriculture a highly vulnerable sector to climate change and variability. As such, major and abrupt changes in the climate system significantly impair the ability of the climate to support agricultural production, consequently threatening global food security (Arora, 2019). Since many of the effects of climate change on agriculture are negative, projections of further climate warming (projected increases of 1.4 to 5.8°C are possible by 2100; Houghton et al., 2001) present a serious concern for the global population.

Viticulture is practiced in regions with specific climate characteristics (low occurrence of freeze events, no incidences of extreme heat, and adequate heat accumulation; White et al., 2006). As such, wine grapes have traditionally been cultivated in regions bounded by the 12 and 22°C mean isotherms during the growing season (Schultz and Jones, 2010). Here, climate plays an important role in regulating grape growth and development, so that wine styles from a specific region possess unique characteristics (Jones, 2015). For instance, solar insolation determines tissue differentiation at bloom and influences sugar levels during ripening (Jones, 2015). Extreme heat during the growing season may halt photosynthesis and berry development, which eventually affects flavor development (Belliveau et al., 2006). Besides, precipitation is needed during specific phases of the crop (e.g., during growth periods) and not so much in others (e.g., at veraison). These delicate climate requirements make viticulture especially vulnerable to climate change, with potential impacts on quantity, quality, and economic viability (Jones et al., 2005; Holland and Smit, 2010). Future climate change will likely have numerous effects on viticulture, namely, altered phenological timing, unbalanced flavor and composition of grapes, and reduced climate suitability for specific varieties grown in some regions (Jones, 2007), among others. Increased risk of pests and diseases is also possible because of significant changes in seasonality and precipitation patterns (Schultze and Sabbatini, 2019). However, climate change is also associated with opportunities, specifically in cool climate viticulture (Mosedale et al., 2015; Schultze et al., 2016b; Jones, 2018). Consistently warmer temperatures in most wine growing regions have necessitated a northward and southward expansion of viticulture (Schultz and Jones, 2010) into areas originally thought to be too cold to support viticulture. Among these is the state of MI, which has warmed enough over the past few decades to support wine grape production.

In the past, viticulture in MI was inhibited by (i) a very short growing season (Schultze et al., 2014, 2016a), (ii) low temperatures during the growing season, (iii) unfavorable precipitation distribution (Schultze et al., 2016a), and (iv) occurrence of very cold winters (Zabadal et al., 2007). Due to this, MI has mostly grown Niagara and Concord juice grapes (Schultze et al., 2014, 2016b) but over time, production costs of these grapes have skyrocketed leaving many MI growers struggling to earn a profit (Schultze et al., 2014). This, coupled with a warming climate, has encouraged a transition to wine grape production. As a result, the amount of land under vinifera cultivation has expanded significantly, such as new development and increases in established vineyards and wineries. This industry has rapidly grown to employ about 28,000 people and generated $2.1 billion in the state by 2017 (John Dunham and Associates, 2017). With such promise, the industry is striving to expand vinifera cultivation to 10,000 acres by 2024 (Hills, 2012). Achieving this goal is challenging, as vinifera is delicately influenced by biophysical conditions (topography and soils) and climate (which is changing significantly). Moreover, despite warming temperatures and, therefore, a generally improving climate for viticulture, some unfavorable conditions exist in MI. The occurrence and seasonal variability of frost events remain largely unchanged, and this continues to threaten viticulture in the region (Schultze et al., 2014). According to the Michigan Craft Beverage Council (n.d.), an estimated 3,050 acres of land is currently under wine grape production. Yet, recent suitability modeling efforts in MI have found that land suitability for viticulture has expanded significantly beyond the traditional growing areas (Wanyama et al., 2020). This increases the need for a more comprehensive understanding of climate factors that are driving the observed expansion in viticultural land suitability as well as those that pose serious threats. Detecting and understanding patterns of long-term trends in these variables can further generate valuable information about current and future changes in MI viticulture and ultimately inform decisions for expanding viticulture in the state.

This study explored the complex climate–viticulture relationship with the goal of advancing knowledge of how climate influences the observed viticultural land suitability expansion in the state of MI. This study, therefore, had two specific objectives. First, it identified major factors influencing land suitability in global viticultural regions (generally) and in the state of MI (specifically). Here, an exhaustive literature review was conducted to provide an in-depth understanding of the main factors influencing viticulture. Second, the study assessed and quantified the nature and magnitude of changes in the identified factors to detect any significant trends in a climate that may be associated with the observed expansion in viticultural suitability in MI. Therefore, the identified variables were analyzed, at multiple scales, to detect patterns and trends over the period 1983–2019. To achieve this, the study used the Mann-Kendall statistical test to assess the presence (or absence) of monotonic trends in the climate variables and Sen’s Slope to quantify the trends. This study was anchored on the assumptions that (i) vinifera has greater expansion capabilities due to the changing climate compared with other agricultural sectors and (ii) the changing climate trends in MI have resulted in more suitable lands for vinifera cultivation over time. The results of this study expand the knowledge of cool-climate agricultural production and suitability for cultivation and highlight the complexity of relating suitability drivers for non-cool-climate to cool-climate vinifera cultivation.

## Study Area, Data, and Methods

### Study Area Description

**Figure 1:**
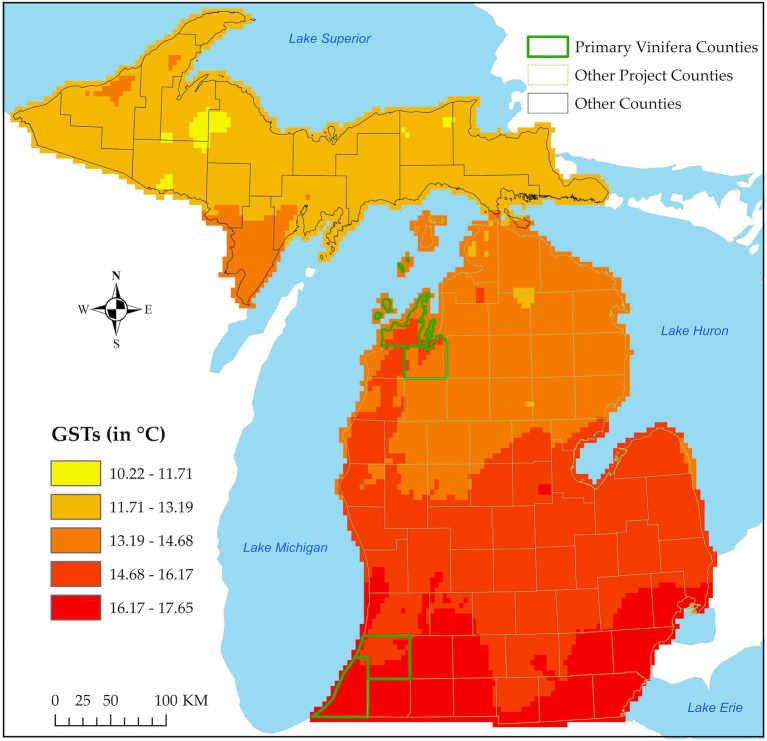
Map of Michigan showing long-term (1983–2019) mean temperatures in the growing season (April–October). Location of the Lower Peninsula counties across the state where trends in climate variables were assessed are outlined in green (traditional counties with vinifera production) and light green (other project counties). Upper Peninsula counties where no trends were assessed are outlined in black.

This study was conducted in the Lower Peninsula of MI in the North Central United States (Figure 1). In this region, a humid continental climate exists, with warm summers, cold winters (Andresen and Winkler, 2009; Perry et al., 2012; Schultze et al., 2014, 2016a,b; Zhuang et al., 2014), and an average growing season temperature (GST) range of 13–15°C (Schultze et al., 2016b). Climate in the region is moderated significantly by the presence of the Great Lakes, with relatively cloudier and milder cold seasons and cooler springs than areas upwind of the lakes (Andresen and Winkler, 2009). Precipitation occurs year-round but is relatively heavier during the warm season and averages 70–80 mm per month (Andresen and Winkler, 2009; Hull and Hanson, 2009; Schultze et al., 2016a). Here, the growing season is defined as the period between budburst and the first fall frost (Schultze et al., 2016b). There also exists a variety of topographic characteristics in MI, such as the hilltops and ridgetops at relatively higher elevations, which help facilitate cold air drainage away from crops and offer some protection against freeze damage (Wanyama et al., 2020). These topographic features, coupled with the effect of the Great Lakes, make it suitable for viticulture. For a more detailed description of the study area, please see Wanyama et al. (2020).

### Data Sources

This study relies on two major data sources: available literature and climate data.

#### Reviewed Literature and Sampling Procedure

An exhaustive literature review was completed to provide an in-depth understanding of major factors influencing global viticultural areas and specifically the observed expansion of vinifera suitability in the cool-climate region of MI under climate change. Literature was gathered from journal publications, university websites, and grape-grower handbooks describing the interrelationships between climate and wine grape production, and consequently land suitability for the cultivation of vinifera in MI. Major controlling factors, most of which are discussed in the previous study by Wanyama et al. (2020), include:

- Climate: including growing degree days (GDDs), frequency of cold days (FCDs), frost-free days (FFDs), spring temperatures, GSTs, winter temperatures, late spring and early fall frost, mean monthly and seasonal precipitation, and number of wet days per month (>3 mm).
- Soils: including soil drainage, depth of rooting zone, and soil pH and depth to bedrock.
- Topography: including slope, slope aspect, and sinks.
- Land cover: land use

The study was specifically interested in how the factors influenced vinifera varietals common to MI. These articles were gathered from both Web of Science^1^ and Google Scholar^2^ using both general searches (e.g., “Viticulture” and “Climate Change”) and very specific searches (e.g., “Riesling” and “GDDs”). The relevance of each article was assessed, and a total of 44 articles were finally chosen for review in this study. As such, the choice of the papers was heavily based on an extensive literature review and expert knowledge of viticulture and climatology and was centered on major factors influencing main aspects of vinifera production (land suitability, grape phenology, and consequently wine composition and quality). Only articles written in English were selected, and this is acknowledged as a source of uncertainty in the study. The 44 articles included 39 peer reviewed articles, four from university websites, and one from a handbook of a grape grower. No timeline constraint was applied, and the reviewed articles were published between 1972 and 2018. Although it may be argued that some of these are too old to be relevant, viticulture is known to be a very old practice dating back 6,000 years (Schultz and Jones, 2010; Venkitasamy et al., 2019). Besides, viticulture has significantly evolved in MI, from being a primary producer of table grapes (*Vitis labrusca*) in the 1960s to being suitable for cultivation of a wide range of wine grape (*V. vinifera*) varieties (Schultze et al., 2016b) only decades later. As such, these studies are suitable in this one, as they provide a valuable perspective to the changing viticultural landscape in MI. Sixty-one and 32% of the reviewed studies were conducted in North America and Europe, respectively. The remainder was studied from Australia.

#### Climate Data and Variable Computation

This part of the study relied solely on the climate dataset obtained from the Parameter-elevation Regressions on Independent Slopes Model (PRISM Climate Group, 2016) series for the period 1983–2019. This gridded dataset, created from a combination of single site climate observations, digital elevation models, and other spatial data (Daly et al., 2001), was preferred because of its high spatial resolution (4 km), extensive use in previous studies (Jones et al., 2004, 2006, 2010; Nowlin, 2013; Yau et al., 2014; Wanyama et al., 2020), and relatively low errors relative to observations (Nowlin, 2013). Temperature‐ and precipitation-based variables used in the previous viticultural suitability study (Wanyama et al., 2020) were computed from the PRISM data, such as base 10°C GDDs, FCDs, FFDs, spring temperatures, precipitation during critical growth periods, and precipitation amounts when rot is of critical concern. A description of these variables is available in Wanyama et al. (2020). It should be noted that some of these variables (like FCDs) were not common in the reviewed literature. However, based on expert knowledge specific to MI viticulture and climatology, a decision was made to include them in this analysis due to their important influence. In addition, average GSTs and total growing season precipitation were computed. These variables were commonly studied in the reviewed literature as additional factors important in viticulture. Here, total growing season precipitation was computed as the sum of precipitation received between April 1 and October 31 for each year. Average GSTs were calculated as the average temperature over the same period. For reference, ranges for each variable in the Lower Peninsula MI, calculated from PRISM time series (TS) over the study period, are shown in Table 1.

**Table 1:** Typical ranges for each variable in the Lower Peninsula Michigan (MI).

Agroclimatic variable	Typical range	Units
Growing degree days	750–2,040	Accumulated days
Frequency of cold days	0–38	Days
Frost-free days	145–215	Days
Precipitation − growth (total)	0–360	mm
Precipitation – rot (total)	65–630	mm
Spring temperatures (average)	4–16	°C
Growing season precipitation (total)	245–1,090	mm
Growing season temperatures (average)	11–19	°C

These values were calculated from Parameter-elevation Regressions on Independent Slopes Model (PRISM) time series (TS) data over the period 1983–2019. Units for each variable are also provided.

For input into trend analysis models, TS was generated for multiple time scales. More specifically, the TS was generated for each variable for 1983–2019 (entire study period, 37 years), 1983–2002 (first 20 years), 2000–2019 (last 20 years), 1983–1994 (first 12 years), 1995–2006 (middle 12 years), 2007–2019 (last 13 years), 1983–2012 (first 30 years), 1990–2019 (last 30 years), and 2012–2019 (last 8 years). Table 2 below provides a list of the periods and a naming convention used in this study. Performing trend analysis on variables at these time scales was necessary to identify specific time periods when climate conditions necessary for vinifera growth improved or worsened, as a step forward toward explaining the observed expansion in land suitability for vinifera production.

**Table 2:** Time periods considered in trend analysis.

Period	Description
1983–2019	Entire period, 37 years
1983–2002	First 20 years
2000–2019	Last 20 years
1983–1994	First 12 years
1995–2006	Middle 12 years
2007–2019	Last 13 years
1983–2012	First 30 years
1990–2019	Last 30 years
2012–2019	Last 8 years

### Methods

This section describes analyses and methods applied in this study. First, the literature review exercise is discussed, and information extraction is detailed. Next, trend analysis is described.

#### Information Extraction From Literature and Analysis

Selected publications were reviewed and information from and details of the publications reported in form of a survey using the Survey Tool of Qualtrics (2019). Descriptive statistics of the data and key themes from this survey were then compiled. Additionally, pieces of information on how climate, topographic, and soil characteristics influence viticulture both in MI and elsewhere were documented. This is meant to improve the understanding of the fast-evolving MI viticulture industry. This exercise was also meant to identify major climate variables in which persistent trends would be explored in the second part of the study.

#### Trend Analysis: Mann–Kendall and Sen’s Slope

This part of the study borrowed from methodologies from Jones (2012), Bardin-Camparotto et al. (2014), and Deitch et al. (2017). The Mann–Kendall test was performed to assess the presence, or lack thereof, of persistent monotonic trends in each variable TS. This nonparametric test is preferred in many studies because it works well with nonnormally distributed data, which is a major characteristic of most climate data (Jones, 2012). To calculate the Mann–Kendall test statistic, data values are first ordered, evaluated (Khambhammettu, 2005), and each value is then compared with all subsequent values (Khambhammettu, 2005; De Beurs and Henebry, 2010). Initially, the Mann–Kendall S statistic is assumed to be 0, and a value of 1 is added to it if the value of an observation is higher than that of the previous observation (Khambhammettu, 2005; De Beurs and Henebry, 2010). The statistic is reduced by 1 if the value of an observation is lower than the previous observation, and no change is made if the values are equal. The Mann–Kendall test equation is shown in Equation 1 below. Low negative and high positive values of S indicate decreasing and increasing trends, but the strength of the trend is statistically quantified by computing probabilities associated with S and size of the data sample used (Khambhammettu, 2005).

(1) $$S=\overset{n-1}{\underset{k=1}{\sum }}\overset{n}{\underset{j=k+1}{\sum }}sign\left(x_{j}-x_{k}\right)$$

where $sign\left(x_{j}-x_{k}\right)$ = $1\;\;\mathrm{if}\;\;x_{j}-x_{k}>0.$

     = $0\;\;\mathrm{if}\;\;x_{j}-x_{k}\;\;=\;\;0$

     = $-1\;\;\mathrm{if}\;\;x_{j}-x_{k}<\;\;0$

     Source: (Khambhammettu, 2005)

This study also used the Sen’s Slope estimator (Sen, 1968) to quantify the magnitude of the trends, which represents the long-term rate of change per unit time (Bardin-Camparotto et al., 2014). These algorithms were used to detect and characterize any persistent trends in important climate variables. From this, a theoretical understanding can be developed of how persistent changes in each variable may be contributing to (or inhibiting) the observed (or potential) spatiotemporal expansion in vinifera suitability in MI. All tests in this study were performed at the 10% significance level.

## Results and Discussion

Results from both the literature review survey and trend analysis are presented in this section. First, descriptive statistics of the surveyed literature are presented. Further, major factors influencing land suitability across the globe, as well as wine composition and quality, are explained. Trend analysis results are also presented and essentially highlight portions of MI that experienced persistent changes in temperature‐ and precipitation-based variables. Information is then synthesized to explain the expanding viticulture industry in MI.

### Descriptive Statistics From the Literature Review

The survey revealed that climate variables were very commonly studied as major factors shaping the viticultural landscape across the globe. Among these, variables derived from temperature were the most popular with GDD, GSTs, and winter temperatures, respectively, appearing in over 56, 47, and 43% of the reviewed literature. Number of FFDs, which determines the length of the growing season in cool climate regions (Schultze et al., 2016b), was studied in over 30% of the reviewed literature. Late spring and early fall frost and FCDs were the least studied temperature variables, both of which appeared in 5 (11%) of the reviewed studies. The second climate variable, precipitation, was also popular, with mean seasonal precipitation studied in 12 (27%) of the reviewed articles. The second category of commonly studied variables was soils, with soil drainage appearing in 25% of the studies.

### Key Messages: Factors Influencing Vinifera Production

This part of the study discusses major factors influencing vinifera production in viticultural regions around the globe (generally) and in MI (specifically). It attempts to explain the observed expansion of land suitability in MI by drawing from literature published about viticulture in the region and elsewhere.

#### Land Suitability for Viticulture

Viticulture is an old practice dating back over 6,000 years (Schultz and Jones, 2010; Venkitasamy et al., 2019), and the quality of produced wine depends on multiple factors, such as climate, soil, and wine making techniques (Ollat et al., 2016). However, the effect of climate is greatest (Van Leeuwen et al., 2004) and therefore, due to limiting climate requirements for vinifera growth, viticulture is largely practiced in regions with specific climate characteristics (Schultz and Jones, 2010): areas with low risk of frost occurrence, adequate heat accumulation, and minimal extreme heat incidents (White et al., 2006). As a result, the best wines of the world come from regions with a certain balance among the three climate attributes (Schultz and Jones, 2010), traditionally found within 30–50°N and 30–40°S latitudes (Jones, 2003; Nesbitt et al., 2018). However, many recent studies have reported an observed warming trend in most viticultural regions (Jones, 2005; Neethling et al., 2012; Santos et al., 2012; Bardin-Camparotto et al., 2014; Kryza et al., 2015). A similar warming trend has been reported in MI (Schultze et al., 2014, 2016b; Schultze and Sabbatini, 2019) and has been linked with the observed expansion in land suitability for both wine and table grapes (Schultze et al., 2016b; Wanyama et al., 2020). The warming climate is evidenced by increased GDD accumulations in the region (Schultze et al., 2014, 2016b; Schultze and Sabbatini, 2019).

Many climate variables influence the suitability of a region for vinifera growth. Perhaps, the most important variables are temperature-related (Nowlin, 2013), namely, accumulated GDDs, GSTs, winter temperatures, number of FFDs, the occurrence of late spring and early fall frost, and FCDs. Accumulated GDDs are a temperature-based proxy for thermal time and have been used in many studies to describe temperature-dependent rates of crop growth and development and seasonal thermal requirements, such as grouping regions with varying levels of suitability (Meinert and Busacca, 2000). GDDs are usually calculated as the sum of temperatures above a base threshold temperature during a period of interest. For viticultural applications, the summation period is typically the growing season with a base temperature of 10°C (Olsen et al., 2011; Anderson et al., 2012; Jones, 2012; Neethling et al., 2012; Modica et al., 2014; Schultze et al., 2014; Badr et al., 2018a), below which vinifera growth is presumably negligible (Anderson et al., 2012). Neethling et al. (2012) have reported that GDDs are preferred because of their significant correlation with various vine stages [flowering, veraison (beginning of fruit ripening), and harvest]. Generally, a GDDs range of 690–3,000+ is possible for viticultural regions. However, the influence of accumulated GDDs on vinifera depicts significant differences across these regions. For instance, in some regions, like Røsnæs in Denmark, accumulated units above 830 were indicative of high suitability (Olsen et al., 2011), while such values are questionable in areas like Quebec, Canada (Roy et al., 2017), the Pacific Northwest, United States (Badr et al., 2018b), and the Poland-Germany-Czech Republic transboundary region (Kryza et al., 2015). GDDs as high as 2,280–2,480 units are only lowly ranked for vinifera suitability in places like Illinois, United States (Kurtural et al., 2006), and would support only a few vinifera varietals in Washington, United States. Values as high as 2,500–3,000 units favor high production of vinifera, better sugar levels, and ultimately high-quality grapes in the same region (Tukey and Clore, 1972). The number of accumulated heat units is, therefore, key in determining vinifera varietals suited for a specific region. For instance, (Roy et al., 2017) have stated that hybrid vinifera varieties (e.g., Marechal-Foch) do well in places with lower GDDs compared with early-ripening *V. vinifera* (like Pinot Noir). GDDs play a significant role in viticultural suitability especially for cool-cold viticultural regions like MI. Informed by most of the reviewed literature and expert knowledge specific to MI viticulture and climatology, accumulated GDD requirements were varied in the calculation of land suitability for red vs. white vinifera varietals (Wanyama et al., 2020). Consequently, this variation introduced very significant spatial differences in land suitability levels for these varietals across the studied counties. In this region, the GDD–yield relationship is largely positive but can be reversed if GDD accumulations occur too early in the season (Schultze et al., 2016a).

Growing degree day is a great index but not without shortcomings. Some studies have reported its failure to adjust for the increasing day lengths at higher latitudes; and, consequently, it tends to underestimate the grape-producing potential of some viticultural regions (Neethling et al., 2012). Jones et al. (2010) have discussed various methodological issues in GDDs, such as the possibility of other base temperatures applicable to some viticultural regions and differences in the calculation of the index (whether simple degree-day or corn degree-day method). GDDs have also been found to correlate very highly with GSTs, indicating that these two essentially communicate the same information (Jones et al., 2010).

Growing season temperatures have been used severally in assessing the influence of temperature on viticulture. Average GSTs generally define the climate maturity ripening potential for high-quality wine varietals (Jones, 2007; Holland and Smit, 2010), using designations of cool, intermediate, warm, hot, and very hot categories (Jones et al., 2010; Anderson et al., 2012). In this categorization, areas with average GST values lower (greater) than 13°C (24°C) are considered too cool (hot) for vinifera production. In this study, the effect of GSTs (as defined above) and general temperatures during various stages of fruit growth and development (flowering, veraison, and preharvest) were explored. Effect of GSTs on vinifera has been documented in multiple studies. In Washington state and parts of Oregon, United States, areas with GSTs of 18°C were considered highly suitable for vinifera production compared with areas with 13°C (Badr et al., 2018b). In North Carolina, United States, maximum GSTs of 29–32°C are required for photosynthesis (Poling et al., 2007). Caprio and Quamme (2002) concluded that in the Okanagan Valley of British Columbia (Canada), high temperatures greater than 26°C before harvest were associated with good vinifera production, probably because such warm temperatures were critical for fruit quality at harvest and bud initiation and development. In south England, a mean daily temperature below 15°C during the flowering period is considered adverse for vinifera production (Mosedale et al., 2015). Elsewhere, in Australia, higher anthocyanin content in grapes was associated with higher temperatures (Cozzolino et al., 2010), although lower production of anthocyanin is also generally associated with warmer temperatures because of degradation of these pigments at a high level of sugar concentration in the fruit (Cozzolino et al., 2010; Jones, 2015). The positive vinifera-GST association has also been reported in MI (Schultze et al., 2016a). However, higher temperatures in the growing season are not always favorable for vinifera production. It is reported that, while few days of temperatures greater than 30°C enhance the ripening potential of the grapes, prolonged exposure to the temperatures can lead to premature veraison and failure of flavor ripening (Jones, 2015). Extreme temperatures (above 35°C) concern growers because these are known to inhibit photosynthesis (Jones, 2015). For instance, in the Okanagan Valley in Canada, such extreme temperatures “shut down the vine,” thereby delaying berry maturation and sometimes flavor development (Jones, 2015), delaying harvest and increasing the risk of fall frost damage due to an already short growing season.

Winter temperatures (and frequency of cold events) are also a critical factor for vinifera production, especially for regions with continental climates (Jones, 2015). Studies have associated high quality wines with mild winters and therefore low frost damage (Holland and Smit, 2010), and have also shown that vinifera; (i) grows well in areas where coldest temperatures exceed −1.1°C and (ii) can withstand cold temperatures between −5 and −20°C (Jones, 2015). However, the effect of cold temperatures on vines varies by grape variety, time of the season, specific tissue exposed, and the characteristics of the cold temperature episode (e.g., duration of the cold, low temperature attained; Poling et al., 2007). For example, Belliveau et al. (2006) have reported that while temperatures around −18°C cause minor bud and shoot injuries in the Okanagan Valley of Canada, the vine is likely to be killed if it is exposed to these temperatures for more than 4 days. It should also be noted that temperatures lower than −28°C are especially detrimental, since such extremely cold conditions are more likely to severely damage or even kill some vines (Tukey and Clore, 1972). The minimum critical temperatures for vinifera have widely been reported as −23°C, beyond which the crop is likely to suffer mild to serious injury (Tukey and Clore, 1972; Caprio and Quamme, 2002; Belliveau et al., 2006). However, even at these or slightly warmer temperatures, growers can still expect some significant damage to vinifera (Poling et al., 2007). For instance, in the Umpqua Valley of Oregon, United States, areas with temperatures lower than −20.5°C are considered unsuitable for viticulture (Jones et al., 2004), while in central Virginia, growers can expect about 50% injury to buds exposed to −22°C (Poling et al., 2007). It is important to note that frequency of cold events over time matters in viticulture, and the viticultural suitability of an area is significantly reduced with an increased frequency of these events (Kurtural et al., 2006; Poling et al., 2007; due to an increased probability of damage or total loss of the vine). This is key to mention, as a replanted vine often takes 5 years to come into full production (Belliveau et al., 2006).

For cool-climate regions like MI, the growing season is generally defined as the period between budburst and first fall frost (Schultze et al., 2016b). Research has shown that vinifera can be grown in regions with 150–200 + FFDs (Tukey and Clore, 1972; Aney, 1974; Jones et al., 2004; Jones, 2015), and an area with more FFDs coupled with higher temperatures (in the growing season) is especially favorable (Belliveau et al., 2006). FFD requirements vary with vinifera varietal, but an average of 180 FFDs are generally optimal for production (Tukey and Clore, 1972; Aney, 1974; Jones, 2015; Badr et al., 2018b). Some studies have also shown that early ripening varieties require 160–170 FFDs to mature (Aney, 1974), yet this range is only ranked as marginally suitable in other studies (Tukey and Clore, 1972; Aney, 1974). In terms of spring temperatures (and occurrence of late spring frost), studies show that vinifera requires at least 10°C in order to initiate growth (Jones, 2015). Besides, damage to the vine (shoots and buds) is possible with spring temperatures below −1.7°C, depending on the stage of crop development (Shaw, 2005; Belliveau et al., 2006; Poling et al., 2007). Such damage will lead to significantly lower grape yield and quality (Belliveau et al., 2006; Jones, 2015) because once a vine loses its primary buds, it will be dependent on secondary buds, whose fruits mature later with lower tonnage (Belliveau et al., 2006).

The effect of precipitation on agriculture is well-documented. The precipitation–viticulture relationship is a dynamic one. While little evidence exist to suggest upper limits for vinifera production, an annual precipitation of less than 500 mm seems to limit production in some hot climates (Pogue, 2009; Jones, 2015). Since such shortages in precipitation can easily be overcome by irrigation (Pogue, 2009; Lereboullet et al., 2014; Jones, 2015), reduced precipitation does not majorly affect viticulture. However, vinifera production suffers from other important precipitation characteristics, such as its distribution and variability during the growing season. Excess rainfall can negatively influence processes, such as sugar-acidity balancing, flowering, vegetative growth, and disease occurrence (Nesbitt et al., 2018). For example, too much rain is known to hamper pollination at bloom, while during maturation and harvest, it is associated with increased fungal occurrence and swelling of grapes, which dilutes wine flavor and affects yield and quality (Belliveau et al., 2006; Jones, 2015). As such, areas with lower growing season precipitation and lower variability are considered optimal for viticulture (Nesbitt et al., 2018). Besides, grape production is also influenced by precipitation that falls outside of the growing season. For the Okanagan Valley in Canada, a positive association was found between production and January precipitation, especially when the precipitation fell as snow (Caprio and Quamme, 2002). During such cold periods, snow protects grape roots from cold temperatures (Caprio and Quamme, 2002; Shaw, 2005). For the cool climate region of MI, unfavorable precipitation distribution is known to negatively affect vinifera production. In this region, seasonal precipitation peaks during veraison and harvesting periods (Schultze et al., 2016a), which increases the risk of leaf and fruit diseases and failure of the crop to mature fully (Zhuang et al., 2014; Schultze et al., 2016a).

Apart from climate variables, soil and topographic characteristics were found to influence vinifera production. These included soil drainage (25%), slope aspect (23%), and slope (20%). These variables, and others, are not discussed in detail, because this review focuses on climate-related variables influencing viticulture.

#### Climate Change and Viticulture

**Figure 2:**
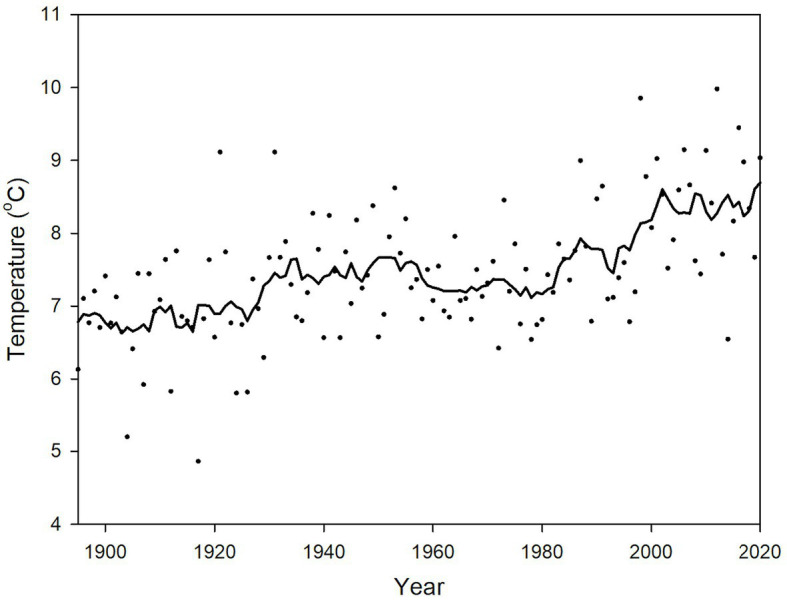
Annual mean temperatures (°C, depicted by dots) spatially averaged across the Lower Peninsula of Michigan, 1895–2020. A 9-year, uniformly weighted moving average is given by the solid black line. Data are based on NOAA NCEI Climatological Division data series (NOAA NCEI, 2007).

**Figure 3:**
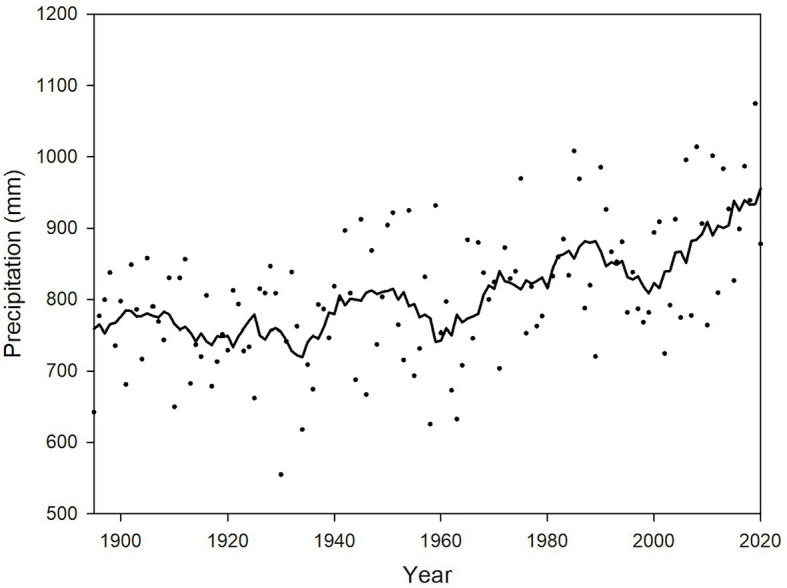
Annual total precipitation (mm) spatially averaged across the Lower Peninsula of Michigan, 1895–2020 (depicted by dots). A 9-year, uniformly weighted moving average is given by the solid black line. Data are based on the NOAA NCEI Climatological Division data series (NOAA NCEI, 2007).

The climate system has been warming in many regions of the world (Jones, 2003; White et al., 2006; Fraga et al., 2012; Schultze et al., 2016b; Nesbitt et al., 2018). This change has introduced both challenges and opportunities for viticulture, such as the rise of new viticultural areas in regions originally thought to be too cold to support wine grape production. Most of these “new world” regions are found in cool climates where climate change effects on viticulture have not fully been investigated (Nesbitt et al., 2018). During the past century, the climate in MI and the surrounding Great Lakes Region has, in general, become warmer and wetter (GLISA, 2019). For reference, the time series of mean annual temperatures and total annual precipitation spatially averaged across the Lower Peninsula of MI from 1895 to 2020 are given in Figures 2, 3, respectively. As can be seen in Figure 2, from the 9-year moving average, there have been notable decadal-scale trends in temperature. There was a period of relatively flat or unchanging mean temperatures during the first 30 years of the series followed by warming temperatures during the 1930s, which was, in turn, followed by a slow cooling trend from 1950 to 1980. During the 1983–2019 study period of interest, mean temperatures warmed by approximately 1°C during the 1980s and 1990s before leveling-off during the last 15–20 years. Overall, mean annual temperatures have increased by approximately 1.5°C during the past century. It is important to note, however, that the observed warming has not been uniform across seasons, with relatively greater changes during the winter and spring seasons and at night with minimum temperatures rising more quickly than daytime maximum temperatures (Andresen et al., 2012; GLISA, 2019). Climate warming over the past few decades places MI in the “zone of transition,” meaning that, over time, climate suitability has expanded to support multiple vinifera varieties (Schultze et al., 2016b). However, besides warming temperatures, other climate-related factors may have also affected vinifera production (see Discussion above). As illustrated in Figure 3, except for some relatively drier years in the late 1950s and early 1960s and again in the late 1990s and early 2000s, mean annual precipitation in Lower MI has increased approximately 150 mm since the late 1930s, resulting in the last complete decade of the study, 2010–2019, as the wettest on record. The increases in precipitation have been associated with both more wet days and greater average precipitation per event with time, such as increases in extreme heavy precipitation events (Andresen et al., 2012). Assessing such notable and persistent changes in these and other variables is, therefore, necessary to develop an understanding of how each is contributing to (or inhibiting) the observed (or potential) spatiotemporal expansion in vinifera suitability in MI.

**Figure 4:**
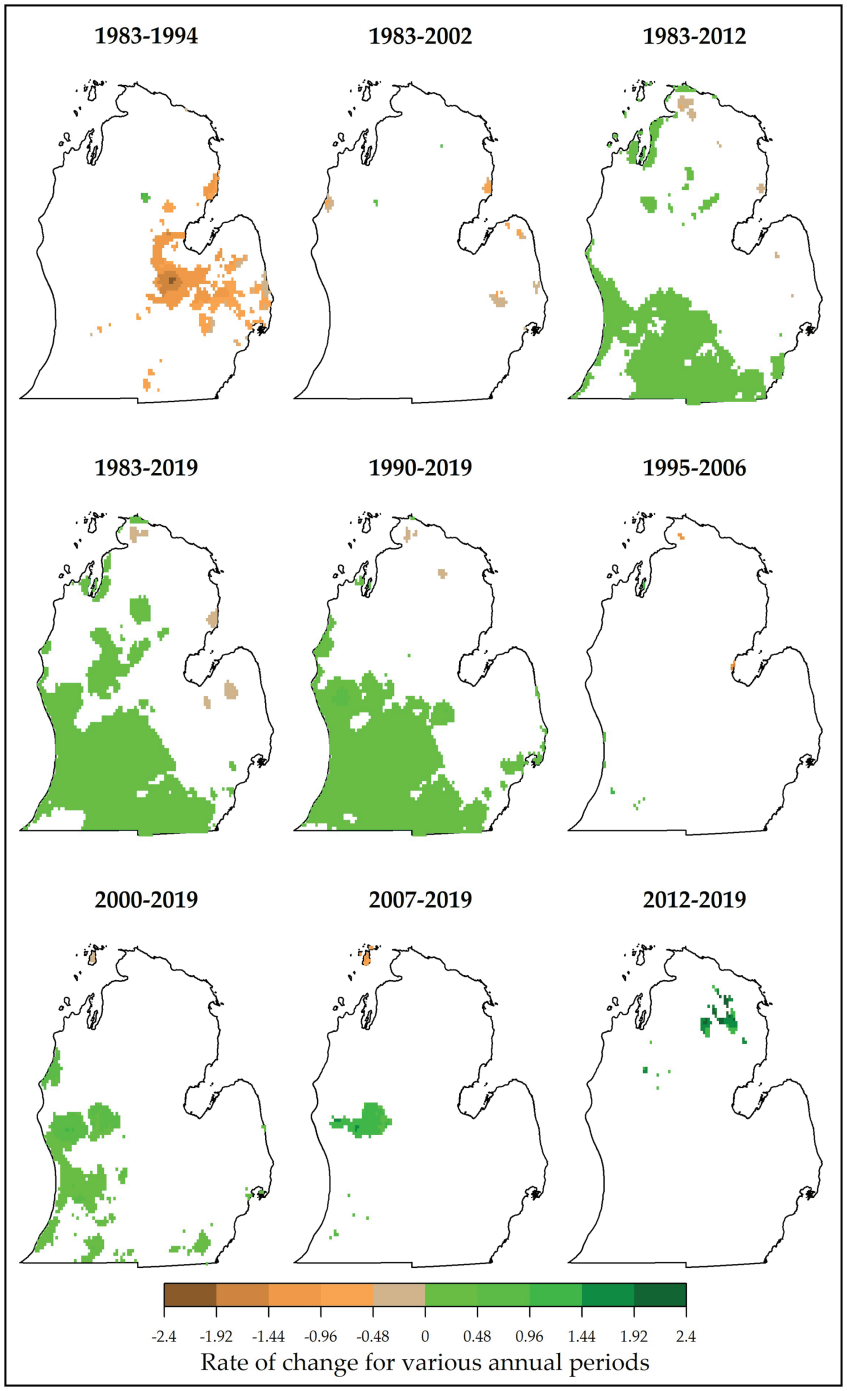
Slope values (Kendall *τ*), indicative of magnitude of change in annual numbers of frost-free days (FFDs; days per year) for different study periods (at *α* = 0.10 level). Highest proportions of changed area were found in 1990–2019, 1983–2019, and 1983–2012. However, greatest changes in FFDs were observed in 1983–1994 (− change) and 2012–2019 (+ change). White pixels indicate no significant change. For reference, typical FFDs in Lower Peninsula MI range from 145 to 215 days per year (Table 1).

Trend analysis revealed persistent changes in climate variables in MI. It was found that, mostly in south and southwestern MI, number of FFDs have significantly increased by up to 0.48 days per year especially in 1983–2012, 1983–2019, and 1990–2019 (Figure 4). Greatest increases (+2.4 days/year) were observed in 2012–2019 in the northern parts of the Lower Peninsula. Largest reductions (up to −2.4 days/year) were observed in the central Lower Peninsula during 1983–2012. Very small proportions of changed area for FFDs were found in 1995–2006, 1983–2002, 2012–2019, and 2007–2019. This result has also been observed in southern Quebec, Canada in which FFDs for Iberville and Granby areas showed strong positive trends (Jones, 2012). Such increases in FFDs are beneficial for cool-climate viticulture.

**Figure 5:**
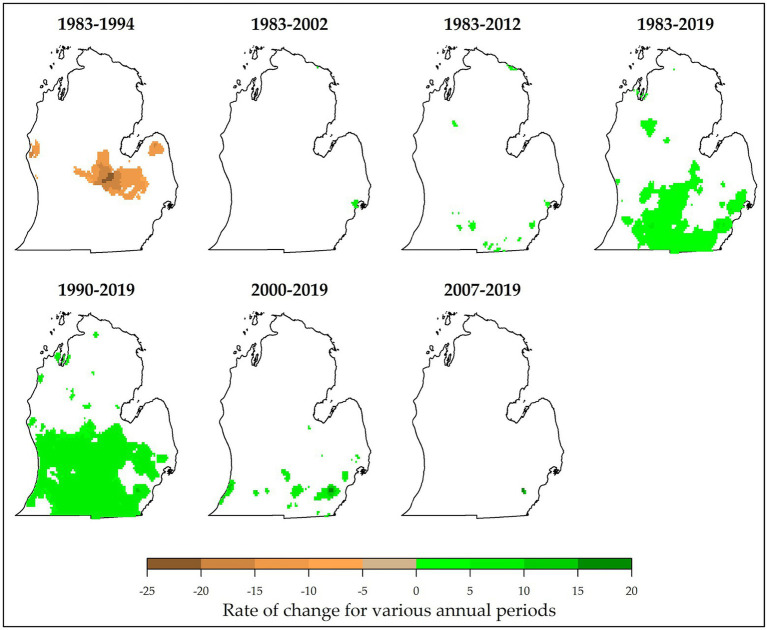
Magnitude of change in the annual numbers of growing degree days (GDDs; accumulated days per year) for different study periods (at *α* = 0.10 level). Locations of persistently changed GDDs are shown. Highest proportions of changed area were found in 1990–2019 and 1983–2019. However, greatest changes in GDDs were observed in 1983–1994 (− change) and 2000–2019 (+ change). For reference, typical GDDs in Lower Peninsula MI range from 750 to 2040 accumulated days per year (Table 1).

Positive trends were also observed in accumulated GDDs, particularly in 1990–2019 and 1983–2019 (Figure 5). An increase of 5–10 GDDs per year was possible for most of the areas in the southern Lower Peninsula. Highest increases in GDDs occurred in 2000–2019 and 2007–2019 (up to +20 GDDs/year). Again, a reduction in GDDs was recorded in 1983–1994 (up to −25 GDDs/year). In terms of proportion, only minimal areas recorded a persistent change in GDDs for 2007–2019, 1983–2002, and 1983–2012. None of the Lower Peninsula showed significant changes in GDDs during 1995–2006 and 2012–2019. Observed and projected increases in accumulated GDDs have been reported in England (Mosedale et al., 2015), Alsace, France (Duchene and Schneider, 2005), Quebec, Canada (Jones, 2012), and MI, United States (Schultze et al., 2014). This increase in GDDs expands suitability for vinifera production, particularly for cool-climate regions. For example, increased GDDs led to more favorable ripening conditions thus higher levels of alcohol content (Duchene and Schneider, 2005). Such increases in accumulated GDDs help explain the expanding climate suitability for vinifera production in MI.

**Figure 6:**
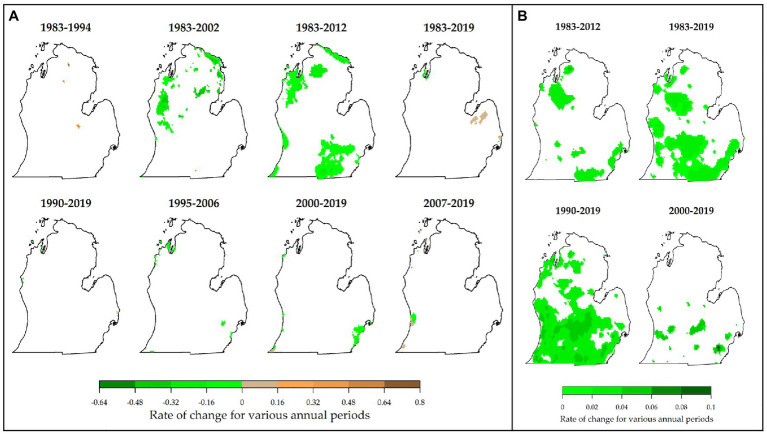
Magnitude of change in the annual frequency of cold days (FCDs; days per year; A) and growing season temperatures (GSTs; B). Locations of persistenly changed FCDs and GSTs are shown.

Fewer areas showed significant changes in FCDs compared with FFDs and GDDs. Persistent changes were observed mostly in 1983–2012 and some in 1983–2002 (Figure 6A). For this variable, a reduction up to 0.16 days/year was common in most of the areas in southern, northern, and northwestern parts of the Lower Peninsula. Highest changes included a decrease up to 0.64 days/year (in 1983–2002) and an increase up to 0.8 days/year (in 1983–1994) but only for a few pixels. Only minimal proportions of the study area showed significant changes in 1983–1994, 1983–2019, 1990–2019, 1995–2006, 2000–2019, and 2007–2019. No significant changes were observed in FCDs in 2012–2019. This result is also consistent with results from a similar study in southwestern MI. Here, Schultze et al. (2014) reported that FCDs changed only slightly, meaning that the quality and quantity of grapes can still be affected by freeze events. Average GSTs showed persistent changes during the 1983–2012, 1983–2019, 1990–2019, and 2000–2019 periods. Here, all areas with significant trends showed warming temperatures, with a majority of them warming by 0.02–0.06°C/year (Figure 6B). Despite this warming during the season, persistent changes in spring temperatures were only observed in the 1983–2002 period (not shown). Positive trends were found in areas mostly located in northern and eastern Lower Peninsula (with increases ranging from 0.36–0.6°C/year). With such warming temperatures in most locations in the Lower Peninsula MI, the production potential for viticulture expands but is greatly threatened by the occurrence of spring temperatures that are not warming in most places.

**Figure 7:**
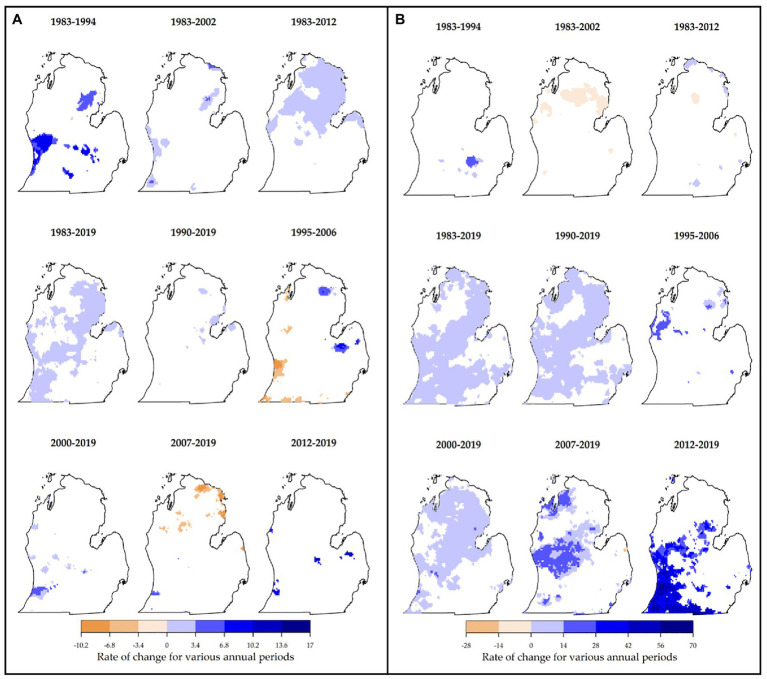
Locations of persistently changed precipitation (mm per year) are shown for key growth periods **(A)** and the general growing season **(B)**. Highest proportions of changed area during key growth periods were found in 1983–2019 and 1983–2012. Greatest changes in the variable were observed mainly in 1983–1994 (+ change). For reference, precipitation during critical growth periods ranges from 0 to 360 mm (Table 1). During the general growing season, negative changes in precipitation were mostly observed in 1983–2002 and 1983–2012. Total growing season precipitation typically ranges from 245 to 1,000+ mm per year (Table 1).

**Figure 8:**
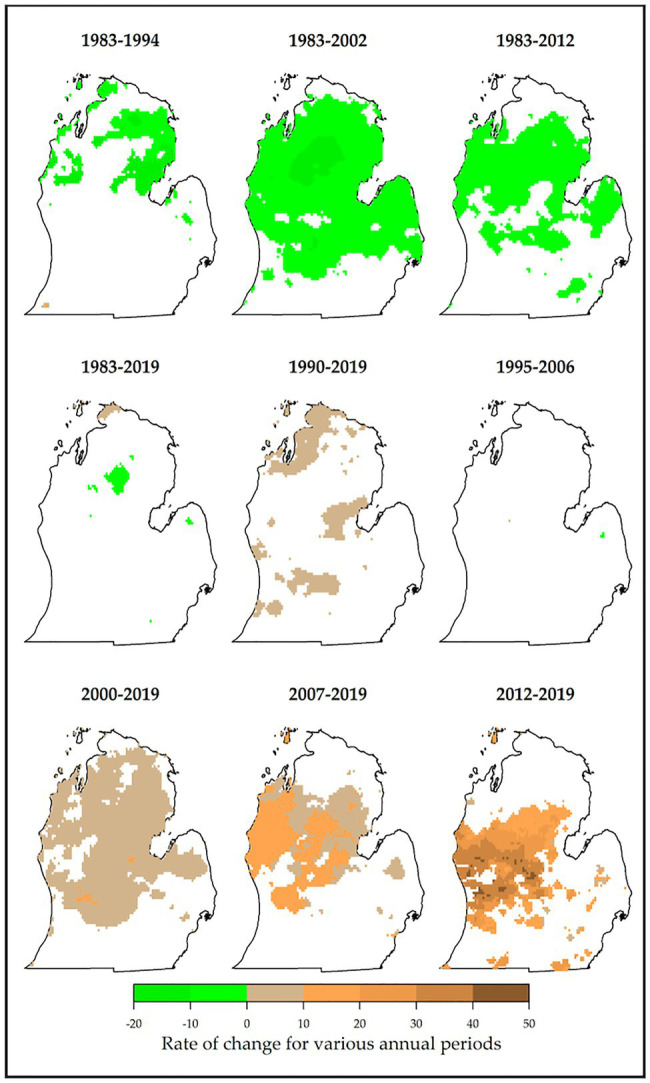
Locations of persistently changed precipitation (mm per year) are shown for critical rot periods. Most of the periods had unidirectional changes (e.g., significant decreases in precipitation in 1983–2002, 1983–2012, and 1983–1994; and increases in 2000–2019, 2007–2019, 2012–2019, and 1990–2019). For reference, precipitation during critical rot periods ranges from 65 to 630 mm (Table 1).

Major trends were observed in precipitation during critical vine growth stages and fruit rot times and the general growing season. During key growth periods, the majority of areas in northern and southwestern Lower Peninsula showed significant increases (particularly for 1983–2012 and 1983–2019, Figure 7A). An increase of up to 3.4 mm/year was common in these periods. Highest increases (+17 mm/year) were experienced in 1983–1994, 1995–2006, and 2012–2019. Significant decreases of 10.2 mm/year were observed in 1995–2006 and 2007–2019. Negligible proportions of changed areas were found in 1990–2019 and 2012–2019. For critical fruit rot times, distinct patterns of change were observed across time periods. There were major decreases in precipitation (range of 0–20 mm/year) for all periods starting in 1983 (Figure 8). This was found in most of the Lower Peninsula especially in 1983–2012 and 1983–2002. However, most periods starting in 1990 or later showed positive increases in precipitation (common increases of 0–20 mm/year). These decreases were observed mostly in northern and western parts of the study area. Lowest proportions of changed areas were found in 1995–2006 and 1983–2019. Looking at the growing season generally, similar patterns were observed. For this variable, both increasing and decreasing trends were detected (Figure 7B), with the latter observed only in 1983–2002 and 1983–2012 (decreases by up to 28 mm/season). Precipitation amounts increased in the rest of the periods in which most of the areas changed by up to 14 mm/year. Greatest changes were observed in 2012–2019 (+70 mm/year) especially in southwestern Lower Peninsula. These results have important implications for MI viticulture. While more precipitation during the growing season promotes crop growth and development, viticulture is threatened by specific precipitation characteristics. For example, more precipitation during key growth periods expands suitability for viticulture as this increase ensures that vines have enough water needed for growth and development. However, the increased precipitation during critical rot periods intensifies the risk of disease and failure of vines to mature fully (Zhuang et al., 2014; Schultze et al., 2016a).

Based on the results and discussion above, the expansion in climate suitability for wine grape production in MI is a function of major changes in climate-related factors. Because of the warming climate, accumulated GDDs and FFDs have increased, while FCDs have reduced slightly over time, thus expanding the ability of this cool climate region to support viticulture. Climate suitability has also expanded because of increases in precipitation during key periods of vinifera growth. However, MI viticulture is still threatened by unfavorable freeze events since only negligible changes have been observed in FCDs (especially in recent years). No significant improvements were found in spring temperatures in recent times, suggesting that late spring freeze events may continue to be a major impediment for viticulture. The increasing precipitation during critical fruit rot periods further inhibits the suitability.

### Importance of Information for the Viticulture Community and Recommendations for the Future

As highlighted throughout the article, grapevine performance is influenced by a wide variety of factors, such as climate variability. In fact, some argue that vinifera is a bio-indicator of global warming (Fernández-González et al., 2013; Biasi et al., 2019). Vine growth, yield, fruit ripening, and composition and, ultimately, wine quality are directly impacted by climate. Further, vineyard impacts due to increased climate variability and change are frequently noted, such as modifications to phenology (Schultz, 2016), impacts to seed dormancy, and change in germination time (Walck et al., 2011). Understanding the driving factors, scales/timeframes of impact, changes in trend over time, and resulting modification from increased climate variability/change is powerful information that can aid in the development of adaptation strategies, management plans, and even expansion plans. Further, studies, such as this, aimed at understanding the consequences of increased climate variability and change is crucial for those in the wine industries to remain competitive as broadening knowledge base and resources can lead to changes and/or better decision-making.

#### Driving Factors

The literature review showed that the driving factors and range of ideal values for such factors were consistent across wine growing regions around the world. Jones et al. (2005) went so far as to say that the wine growing regions of the world are currently at or near their ideal climate for their grape cultivar. MI sticks out a bit in this statement as the wine growing industry is not only thriving but growing in a cool climate. For instance, Keller (2010) noted that, at critical points in vine phenology, optimal temperature ranges for vinifera production varied from 20 to 30°C with temperatures below 15 and above 35°C, leading to marked reductions in yield formation and fruit ripening. MI, rather consistently, falls outside of such ranges but still is a growing producer of wine for the United States. By looking at the trends observed in this article, a clearer picture of vinifera cultivation in MI emerges. Though temperature and precipitation are limiting, over time we have seen a change in climate regimes that favor cultivation across portions of the state. Such a warming trend is seen in several metrics studied, such as GDDs and number of FFDs. Importantly, while these differing factors see this warming trend, the factors themselves impact vinifera cultivation at different times and in different ways. Therefore, highlighting the understanding of the factors and their timing of impact is important for production.

#### Scales of Impact

From the local to farm scale, multiple studies, such as this one, highlight how temperature and water availability (deficit and surplus) are limiting factors in vineyard production (Keller, 2010; Biasi et al., 2019). However, the physiological response of a grapevine to climate is influenced by the soil type, planting density, soil management, pruning, and water management. For instance, water deficit and surplus affect grape composition through inadequate soil available moisture which, at critical points in the phenology of the vine, can impact grape quality (Jackson et al., 1993). Therefore, knowledge of the climate drivers and patterns not only furthers the knowledge of land suitability but also, when the thought of in conjunction with the environmental drivers, gives a broader understanding of the landscape and assists with management decisions. Therefore, understanding the scale of impact for such variables is important information, aiding the growers/wine makers to look at their lands holistically with a spatial and temporal understanding of interconnected drivers of productivity.

#### Change in Trend

Time series data are required to perform a complex trend analysis. Through big data analysis, critical information is extracted to aid growers and the agricultural industry in understanding the seasonal to decadal shifts in driving climatic factors. Understanding trends in climate factors using Mann Kendall and Sen’s slope gives insight into suitability for cultivation through the understanding of change, the magnitude of change, and seasonal variability. With such trend analysis, we can begin to look beyond traditional approaches and think of adaptive management. An increase in the awareness of winegrowers of the need for adaptive management due to climate conditions has been reported (e.g., Biasi et al., 2019). Across the state of MI, there is an active agricultural industry that coordinates with researchers and extension agents to utilize data-driven techniques for landscape management. Through trends seen in this article, and the approaches themselves, we can better assist winegrowers in these efforts, especially because projected increases in climate variability, as noted by the IPCC, fly in the face of attempts of most growers to minimize spatial and annual variability in grape yield and quality. Understanding risk, especially climate risk, and improving preparedness/management to climate impact is an intrinsic property supporting resilience of local agricultural systems (Biasi et al., 2019).

### Study Limitations

This study employed a common literature review methodology employing Web of Science and Google Scholar with the aforementioned search terms. The final 44 articles reviewed are the result of the database review and a double review of each article for facts, data, and results. The only limitation of this approach comes from the databases as their are a wide variety to choose from and different articles are highlighted in each. Web of Science and Google Scholar were selected as they are the most common. The results of the searches were compared between the two databases to: (1) remove duplicates and (2) ensure consistency in the search outcomes. An additional limitation of the study results from the climate data employed. There are a wide variety of gridded climate data readily available to the public for temperature, precipitation, winds, humidity, etc. Each of these datasets has differing resolutions, temporal extent, spatial extent, and interpolation methods employed. PRISM was selected, and as it is very commonly used across the state of MI (e.g., Shao et al., 2015; Wanyama et al., 2020), it is available at a fine enough spatial and temporal extent to calculate the variables needed (e.g., GDDs, FFDs etc.), and it was recommended through personal communication with the State of MI climatologist, Dr. Jeff Andresen. Dr. Andresen suggested PRISM because of results comparing the gridded data with his network of weather stations across the state.^3^

## Conclusion

This study looked to explore the complex relationship between viticulture cultivation and the climate-environmental conditions as it pertains to the state of MI. Through a literature review, the most noted variables impacting viticulture production were identified. Trend analysis on these variables highlights the pattern trajectory of such critical variables both spatially and temporally across the state. The literature review indicates that while MI is a cool climate for grape production, the variables impacting productivity, phenology, harvest, and overall grape quality are similar to those impacting other grape-growing regions around the world, such as, commonly, GDDs. Grape-growing regions are sometimes classified into Winkler regions according to patterns of cumulative GDDs (Amerine and Winkler, 1944). Summation of GDDs across the growing season is standard for the Winkler classification and results in five classifications with the highest (Class 5) having >2,222 GDDs and the lowest (Class 1) having < 1,389 GDDs. Through this study, we show that such classifications with defined ranges and seasonal lengths may miss critical details to viticulture growth in the state of MI. Through trend analysis, we show that changing climate regimes and the timing of such changes are critically important to vinifera cultivation. In addition to GDDs, trend analysis on ground surface temperatures, winter temperatures, the number of FFDs, and precipitation further highlights that MI is in the “zone of transition” for vinifera cultivation. As such, changing climate regimes has, over time, increased the suitability of MI for vinifera cultivation and enabled cultivation to expand to support multiple other vinifera varietals. Winemaking across the state of MI is actively expanding as wine-growing groups, agriculture experts, and others push for industry expansion. By combining the literature review with the trend analysis, we are developing a robust means for growers to assess the suitability of land for cultivation and understand the drivers of suitability, both now and into the future. Using these variables, trends, and expert knowledge, spatially explicit models of suitability were developed by varietal (Wanyama et al., 2020). Further, with such suitability modeling and detailed knowledge of the spatial trends and patterns of variables driving vinifera cultivation, we can develop forecasts for climate impacts to the industry. Ultimately, this study looks to develop meaningful data and analysis (patterns and trends) for the viticulture community across the state of MI to help inform decision-making processes.

## Data Availability Statement

Publicly available datasets were analyzed in this study. This data can be found here: parameter-elevation regressions on independent slopes model (PRISM Climate Group, 2016).

## Author Contributions

This article was written collaboratively with a group of faculty and academic staff at Michigan State University. EB and RG: conceptualization. EB and DW: methodology. DW: formal analysis. RG, DW, and NW: data curation. EB, DW, and JA: writing. EB, DW, PS, and JA: review and editing. DW and JA: visualization. EB: supervision, administration, and funding acquisition. All authors contributed to the article and approved the submitted version.

### Conflict of Interest

The authors declare that the research was conducted in the absence of any commercial or financial relationships that could be construed as a potential conflict of interest.

## Footnotes

^1^www.webofknowledge.com

^2^https://scholar.google.com

^3^https://enviroweather.msu.edu/
